# Canonical NF-κB signaling pathway and GRO-α/CXCR2 axis are activated in unruptured intracranial aneurysm patients

**DOI:** 10.1038/s41598-022-25855-2

**Published:** 2022-12-09

**Authors:** Joanna Kamińska, Marzena Tylicka, Violetta Dymicka-Piekarska, Zenon Mariak, Joanna Matowicka-Karna, Olga Martyna Koper-Lenkiewicz

**Affiliations:** 1 Department of Clinical Laboratory Diagnostics, Medical University of Białystok, 15A Jerzego Waszyngtona St., 15-269 Białystok, Poland; 2 Department of Biophysics, Medical University of Białystok, Białystok, Poland; 3 Department of Neurosurgery, Medical University of Białystok, Clinical Hospital of the Medical University of Białystok, Białystok, Poland

**Keywords:** Neurology, Neurological disorders, Diagnostic markers, Diseases of the nervous system

## Abstract

Activation of the nuclear factor kappa-B (NF-κB) stimulates the production of pro-inflammatory molecules involved in the formation of intracranial aneurysms (IA). The study aimed to assess the NF-κB p65 subunit and the GRO-α chemokine and its receptor CXCR2 concentrations in unruptured intracranial aneurysm patients (UIA, n = 25) compared to individuals without vascular changes in the brain (n = 10). It was also analyzed whether tested proteins are related to the size and number of aneurysms. Cerebrospinal fluid (CSF) and serum protein levels were measured using the ELISA method. Median CSF and serum NF-κB p65 concentrations were significantly lower, while median CSF GRO-α and CXCR2 concentrations were significantly higher in UIA patients compared to the control group. CSF and serum NF-κB p65 concentrations negatively correlated with the number of aneurysms. In UIA patients the median GRO-α concentration was two-fold and CXCR2 almost four-fold higher in CSF compared to the serum value. CSF GRO-α concentration positively correlated with the size of aneurysms.Significantly decreased CSF NF-κB p65 and significantly increased CSF GRO-α and its CXCR2 receptor concentrations in UIA patients compared to the control group may altogether suggest that the canonical NF-κB signaling pathway is activated and its target pro-inflammatory genes are highly expressed in UIA patients. However, to unequivocally assess the involvement of the classical NF-κB pathway with the participation of the NF-κB p65 subunit and the GRO-α/CXCR2 axis in the formation of IA, further in vivo model studies are needed.

## Introduction

Hemodynamic changes and inflammation in the cerebral vessel are two of the key factors responsible for the formation and development of intracranial aneurysms (IA)^1–5^. According to some authors, the first step initiating the formation of IA is the activation of the nuclear factor kappa B (NF-κB) as a result of hemodynamic stress (shear stress) and pro-inflammatory cytokines acting on the cerebral artery wall^6–10^. This hypothesis is supported by the bioinformatics analyses conducted by Poppenberg et al., who in patients with unruptured intracranial aneurysms (UIA) showed that among the genes allowing to distinguish patients with cerebral aneurysms with a diagnostic accuracy of 85% from those without vascular changes in the brain, there were genes strictly related to the NF-κB pathway^11^.

NF-κB (nuclear factor kappa-light-chain-enhancer of activated B cells) is a protein complex that acts as a transcription factor. The NF-κB family of proteins consists of two classes. Class I includes NF-κB1 (alias p105, p50) and NF-κB2 (alias p100, p52). Class II includes RelA (alias p65), RelB, and c-Rel. NF-κB signaling acts through two interconnected pathways classical/canonical and alternative/non-canonical, which differ in both signaling components and biological functions^12–14^. Activation of the classical NF-κB pathway leads to a chronic inflammatory response, including the induction of pro-inflammatory cytokine expression, e.g. GRO-α/chemokine C-X-C motif ligand 1 (CXCL1) chemokines^15,16^, thus being responsible for the formation and development of brain aneurysms. In vitro, GRO-α provides leukocyte chemotactic activity and endothelial cell proliferation^17,18^. In addition, GRO-α as an α-chemokine with the ELR + motif (Glu-Leu-Arg) promotes angiogenesis^19^.

Single studies assessing the role of NF-κB in the formation of brain aneurysms are available in the literature^6,12,20,21^. These are studies in animal models of IA, indicating activation of the RelA subunit (p65) and increased expression of the NF-κB p50/p65 heterodimer in endothelial cells and macrophages^1,6,10,12^. Unfortunately, some of the studies conducted do not even specify which NF-κB subunit was analyzed^21^. Therefore, the study aimed to assess the concentration of the NF-κB p65 subunit and the GRO-α chemokine and its C-X-C Motif Chemokine Receptor 2 (CXCR2) in patients with UIA compared to those without vascular changes in the brain. It was also analyzed whether the concentrations of the studied proteins NF-κB p65, GRO-α, and CXCR2 depend on the size and number of aneurysms in patients with UIA.

## Results

Detailed median values with 25th and 75th percentiles of proteins tested in unruptured intracranial aneurysm (UIA) patients and the control individuals without vascular lesions in the brain are presented in supplementary files (Supplementary Table S1).

### NF-κB p65 signaling pathway is activated in unruptured intracranial aneurysm patients

**Figure 1:**
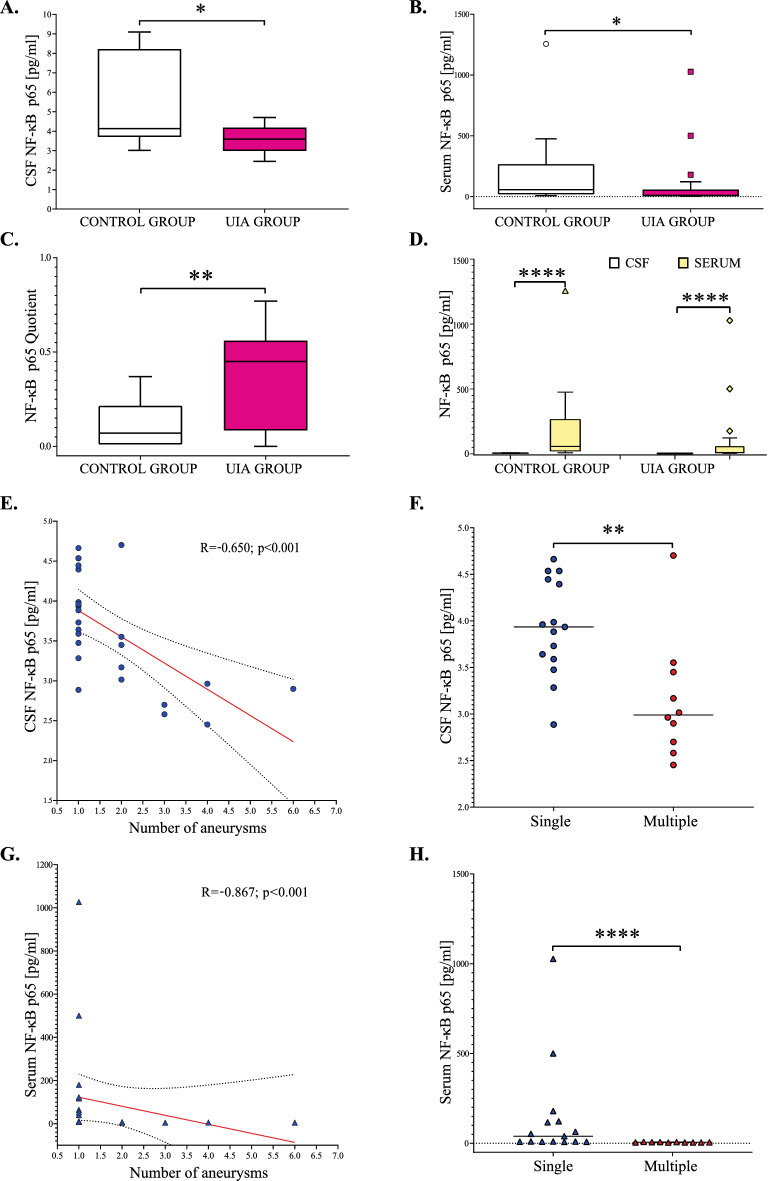
(**A**–**H**) Cerebrospinal fluid, serum, and Quotient results for NF-κB p65 in unruptured intracranial aneurysm patients and the control group. The Quotient was calculated by dividing the CSF protein value by the serum protein value. Statistical significance: * p < 0.05, ** p ≤ 0.01, **** p ≤ 0.001. CSF, Cerebrospinal fluid; NF-κB p65, nuclear factor kappa-B p65 subunit; UIA, unruptured intracranial aneurysm.

Median cerebrospinal fluid (CSF) and serum NF-κB p65 concentrations in UIA patients were significantly lower, while the median NF-κB p65 Quotient was significantly higher compared to the control group (p = 0.03, p = 0.02, and p = 0.01, respectively) Fig. 1A–C. In both, the study and the control groups, CSF NF-κB p65 levels were significantly lower compared to the serum values (p < 0.0001, respectively) Fig. 1D. We also found that CSF as well as serum NF-κB p65 concentrations negatively correlated with the number of aneurysms. Further analysis revealed, that patients with multiple brain aneurysms had significantly lower CSF and serum NF-κB p65 levels compared to individuals with single brain aneurysms (p = 0.003 and p < 0.0001, respectively) (Fig. 1E–H).

### GRO-α and CXCR2 are synthesized intrathecally in unruptured intracranial aneurysm patients

**Figure 2:**
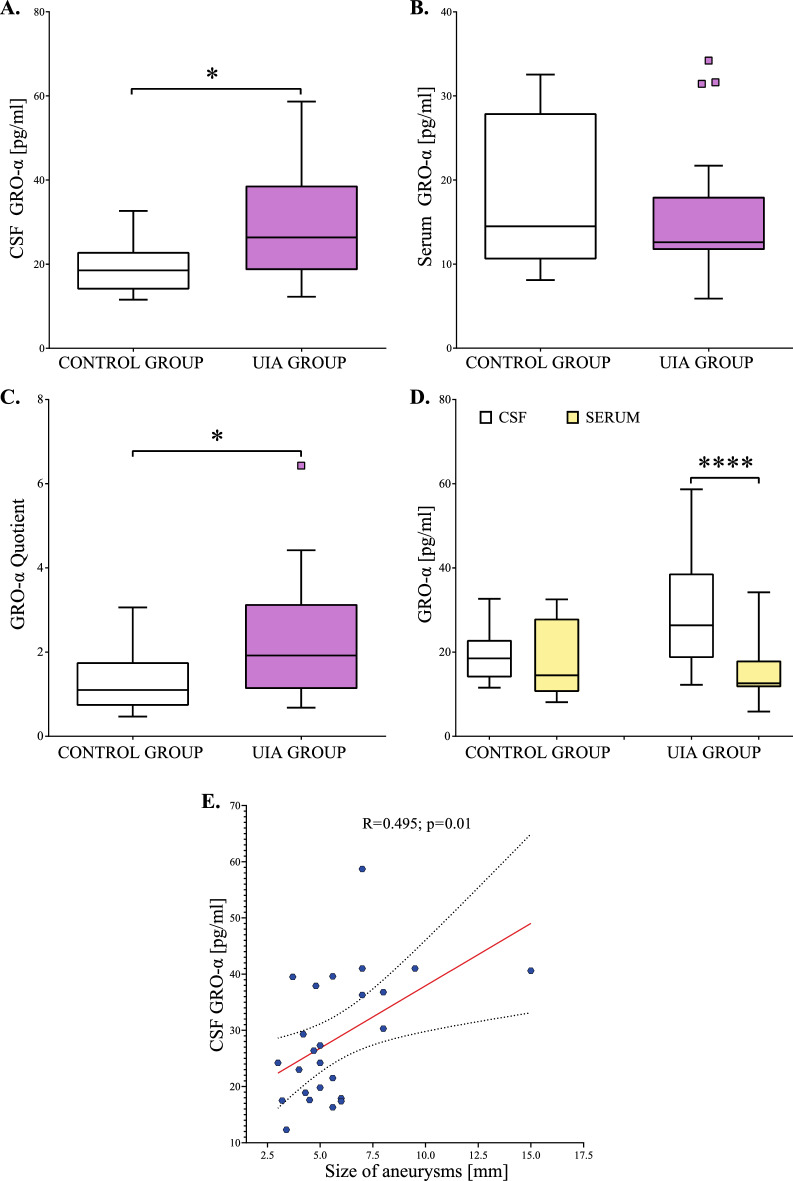
(**A**–**E**) Cerebrospinal fluid, serum, and Quotient results for GRO-α in unruptured intracranial aneurysm patients and the control group. The Quotient was calculated by dividing the CSF protein value by the serum protein value. Statistical significance: * p < 0.05, **** p ≤ 0.001. CSF, Cerebrospinal fluid; GRO-α, GRO alpha chemokine; NF-κB p65, nuclear factor kappa-B p65 subunit; UIA, unruptured intracranial aneurysm.

Median CSF GRO-α concentration was significantly higher in UIA patients compared to the control group (p = 0.01) Fig. 2A. Median serum GRO-α concentration in UIA individuals was a little lower than in the control group, but the difference was not significant (p = 0.69) Fig. 2B. Median GRO-α Quotient was significantly higher in UIA patients compared to the control group (p = 0.04) Fig. 2C. Moreover, in UIA patients median CSF GRO-α concentration was two-fold higher compared to the median serum concentration (p < 0.001). In the control group, we did not observe such a difference (p = 0.10) Fig. 2D. We also found that CSF GRO-α concentrations positively correlated with the size of aneurysms (Fig. 2E).

**Figure 3:**
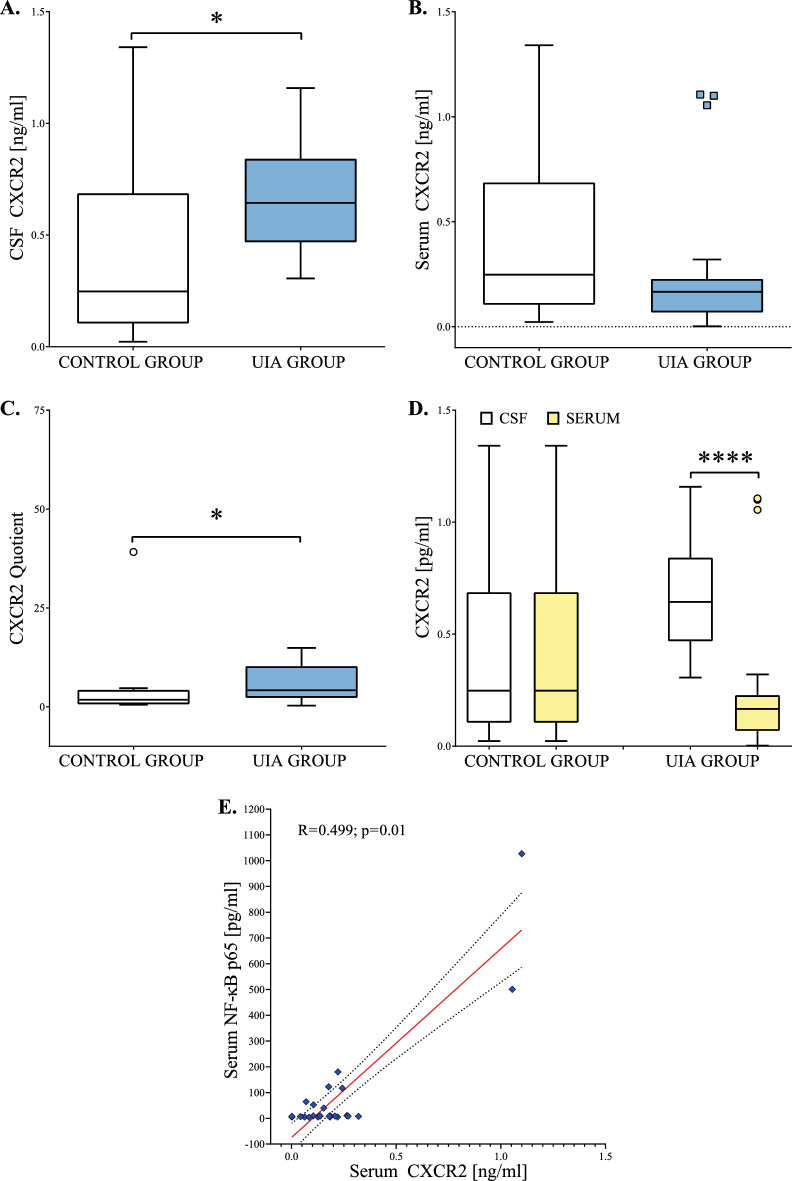
(**A**–**E**) Cerebrospinal fluid, serum, and Quotient results for CXCR2 in unruptured intracranial aneurysm patients and the control group. The Quotient was calculated by dividing the CSF protein value by the serum protein value. Statistical significance: * p < 0.05, **** p ≤ 0.001. CSF, Cerebrospinal fluid; CXCR2, C-X-C Motif Chemokine Receptor 2; UIA, unruptured intracranial aneurysm.

Median CSF CXCR2 concentration was significantly higher in UIA patients compared to the control group (p = 0.03) (Fig. 3A). Median serum CXCR2 concentration in UIA individuals was a little lower than in the control group, but the difference was not significant (p = 0.11) (Fig. 3B). Median CXCR2 Quotient was significantly higher in the UIA group compared to the control individuals (p = 0.03) (Fig. 3C). Moreover, in UIA patients median CSF CXCR2 concentration was almost four-fold higher compared to the serum median value (p < 0.001). In the control group, we did not observe such a difference (p = 0.10) (Fig. 3D). We also found that serum CXCR2 concentrations positively correlated with serum NF-κB p65 concentrations (Fig. 3E).

### NF-κB p65, GRO-α, and CXCR2 Quotients calculation is useful in differentiating UIA patients from individuals without vascular lesions in the brain

**Figure 4:**
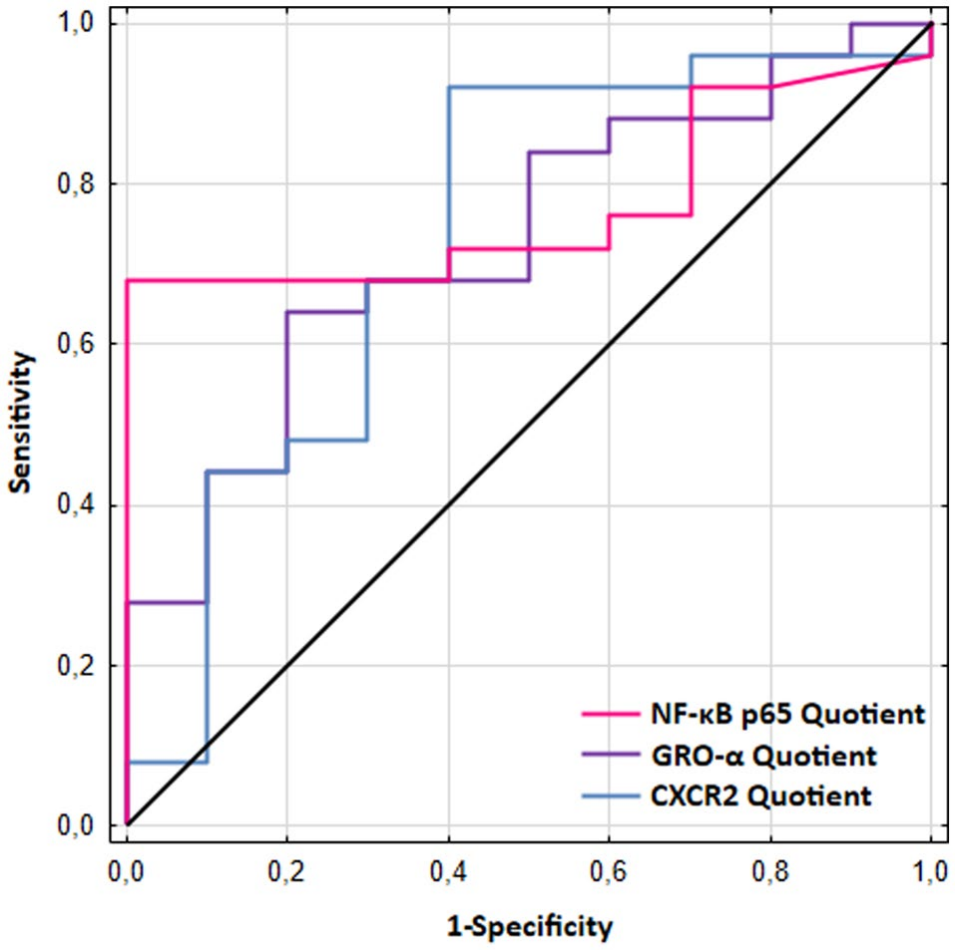
Areas under the ROC curves (AUCs) for NF-κB p65, GRO-α, and CXCR2 Quotients in differentiating unruptured intracranial aneurysm patients from individuals without vascular lesions in the brain. Quotients were calculated by dividing the CSF protein value by the serum protein value. For NF-κB p65 Quotient the AUC = 0.772, cut-off = 0.37; for GRO-α Quotient the AUC = 0.728, cut-off = 1.60; for CXCR2 Quotient the AUC = 0.732, cut-off = 2.07.

NF-κB p65 Quotient was the most useful in differentiating UIA patients from individuals without vascular lesions in the brain. It revealed the highest AUC (0.772), diagnostic specificity (100%) as well as positive predictive value (100%). The remaining AUCs were also statistically higher than AUC = 0.5, which indicates their diagnostic usefulness for differentiating patients with UIA from subjects without vascular lesions in the brain (Table 1, Fig. 4).

**Table 1:** Diagnostic usefulness of NF-κB p65, GRO-α, and CXCR2 evaluation in differentiating unruptured intracranial aneurysm patients from individuals without vascular lesions in the brain.

	Cut-off	Youden index	AUC ± SE	Se [%]	Sp [%]	PPV [%]	NPV [%]	ACC [%]		2-tailed p-value
CSF NF-κB p65, pg/ml	3.73	0.40	0.734 ± 0.094	60	80	88	44	66	0.01	
Serum NF-κB p65, pg/ml	10.63	0.58	0.760 ± 0.081	68	90	94	53	74	0.001	
NF-κB p65 Quotient	0.37	0.58	0.772 ± 0.078	68	100	100	56	77	< 0.001	
CSF GRO-α, pg/ml	23.00	0.44	0.760 ± 0.085	64	80	89	47	69	0.002	
GRO-α Quotient	1.60	0.44	0.728 ± 0.091	64	80	89	47	69	0.01	
CSF CXCR2, ng/ml	0.31	0.60	0.736 ± 0.119	100	60	88	100	90	0.047	
CXCR2 Quotient	2.07	0.52	0.732 ± 0.103	92	60	85	75	83	0.02	

ACC, diagnostic accuracy; AUC, area under the ROC curve; CSF, Cerebrospinal fluid; Cut-off (based on the highest Youden index); CXCR2, C-X-C Motif Chemokine Receptor 2; GRO-α, GRO alpha chemokine; NF-κB p65, nuclear factor kappa-B p65 subunit; NPV, negative predictive value; PPV, positive predictive value; SE, Standard Error; Se, diagnostic sensitivity; Sp, diagnostic specificity.

## Discussion

In our study, for the first time in patients with unruptured intracranial aneurysms (UIA), we assessed the concentration of the transcription factor NF-κB p65 in the cerebrospinal fluid (CSF) and the serum. We showed that the concentration of NF-κB p65 in both materials was significantly lower in patients with UIA compared to those without vascular changes in the brain and significantly lower in CSF compared to the serum of these patients. Decreased NF-κB p65 subunit concentration, especially in the CSF, may suggest its increased activation within the central nervous system (CNS), thus insinuating its potential role in the formation of intracranial aneurysms in humans.

The NF-κB proteins contain nuclear localization sequences that in an inactive state are blocked by linked inhibitors of the IκB family. NF-κB activation occurs through the phosphorylation and degradation of IκB (via IKK2 kinase). After IκB detachment, NF-κB proteins immediately migrate to the nucleus where they bind to DNA to act as transcription factors^22,23^. Classical activation of the NF-κB p50-p65 and c-Rel heterodimer leads to rapid but transient activation of NF-κB acting as a strong activator of target gene transcription^24,25^. In turn, homo- and heterodimers of NF-κB1 (p105) and NF-κB2 (p100) subunits suppress the transcription of NF-κB target genes^13,26^.

Aoki et al., in a mouse model study, demonstrated increased activation of the NF-κB p65 subunit to form the NF-κB p50/p65 heterodimer in the aneurysmal wall of an early stage of IA formation. In addition, the authors showed that the deficiency or inhibition of the NF-κB p50 subunit by decoy oligonucleotides or anti-NF-κB molecules contributes to the arrest of IA formation and progression by suppressing the production of chemokines, interleukins, and matrix metalloproteinases^6,10^. Also, Liu et al. showed increased expression of NF-κB in the early development of cerebral aneurysms^12^.

Xian-Liang et al., in immunohistochemical studies in a mouse model showed increased expression of the NF-κB p65 subunit in the wall of unruptured and ruptured IA. The authors also noted that activation of the NF-κB p65 signaling pathway was correlated with larger aneurysm sizes^20^. It is worth noting that in our study we showed a strong negative correlation of NF-κB p65 concentrations with the number of aneurysms and we found that patients with multiple aneurysms had significantly lower concentrations of NF-κB p65 compared to those with single aneurysms. These data together may suggest that the canonical NF-κB p65 pathway could participate in the formation and progression of brain aneurysms.

Increased activity of NF-κB leads to enhanced expression of cell adhesion molecules, pro-inflammatory cytokines, or ELR+ chemokines such as CXCL8/IL-8 and CXCL1/GRO-α in the vascular endothelium^27^. In response to stimulation with pro-inflammatory cytokines, also microglia and astrocytes may secrete GRO-α^28^, which then binds to the CXCR2 receptor on the surface of the vascular endothelium, leading to its activation with subsequent recruitment of leukocytes^29^. A cascade of these events can contribute to the development of IA.

In our study, we were the first to assess the concentration of the GRO-α chemokine and its CXCR2 receptor in both CSF and serum of patients with UIA. We have demonstrated a significantly higher concentration of the GRO-α chemokine and its CXCR2 receptor in CSF of patients with UIA compared to those without vascular changes in the brain. It should be especially emphasized that in UIA patients the concentration of the GRO-α chemokine was two-fold higher, and the concentration of its CXCR2 receptor was almost four-fold higher in CSF compared to the serum concentration. We did not observe such differences in the control group, which suggests that in UIA patients, both the GRO-α chemokine and its receptor are synthesized locally within the CNS. On this basis, we conclude that the GRO-α/CXCR2 axis may play a significant role in the formation of IA, and the source of the increased concentration in CSF of GRO-α and its CXCR2 receptor may be increased activation of the classical NF-κB pathway with the participation of NF-κB p65 subunit. Especially, since we showed a significant relationship between CXCR2 and NF-κB p65 in UIA patients. However, to ultimately indicate the involvement of the classical NF-κB pathway with the participation of the NF-κB p65 subunit and the GRO-α/CXCR2 axis in the formation of IA, further in vivo model studies are needed.

The role of GRO-α in the development and progression of IA may be mainly related to the chemotaxis of neutrophils that release pro-inflammatory cytokines^30^. PMN-I pro-inflammatory neutrophils secrete IL-12 and MIP-1α, while PMN-II inflammatory neutrophils secrete IL-10 and MCP-1. Then PMN-I polarizes macrophages to the pro-inflammatory phenotype M1, which synthesizes cytokines such as IL-1, -6, -8, -12, -23, TNF-α, and PMN-II polarizes macrophages to the M2 phenotype^31^. Research by Nowicki et al. in a mouse model showed that GRO-α-dependent neutrophil infiltration is of key importance in modulating the macrophage M1 phenotype, and blocking GRO-α inhibited the formation of IA in mice by significantly reducing neutrophil infiltration^32^. The authors additionally emphasize that they did not observe the expression of the GRO-α chemokine in a normal artery, but only in murine aneurysms^7^.

Our previous research showed that the calculation of Quotients for IL-6 and IL-8 appears to be more clinically useful for indicating individuals with UIA compared to the analysis of CSF or serum protein levels separately^4,5^. Thus in the current study for proteins tested we also calculated their Quotients. We showed, that NF-κB p65 Quotient was the most useful in differentiating UIA patients from individuals without vascular lesions in the brain. However, AUCs for both GRO-α Quotient and CXCR2 Quotient were also statistically higher than AUC = 0.5, which indicates their diagnostic usefulness for differentiating patients with UIA from subjects without vascular lesions in the brain. These results indicated, that the calculation of proteins Quotients seems to be clinically useful for indicating individuals with UIA.

## Conclusions

**Figure 5:**
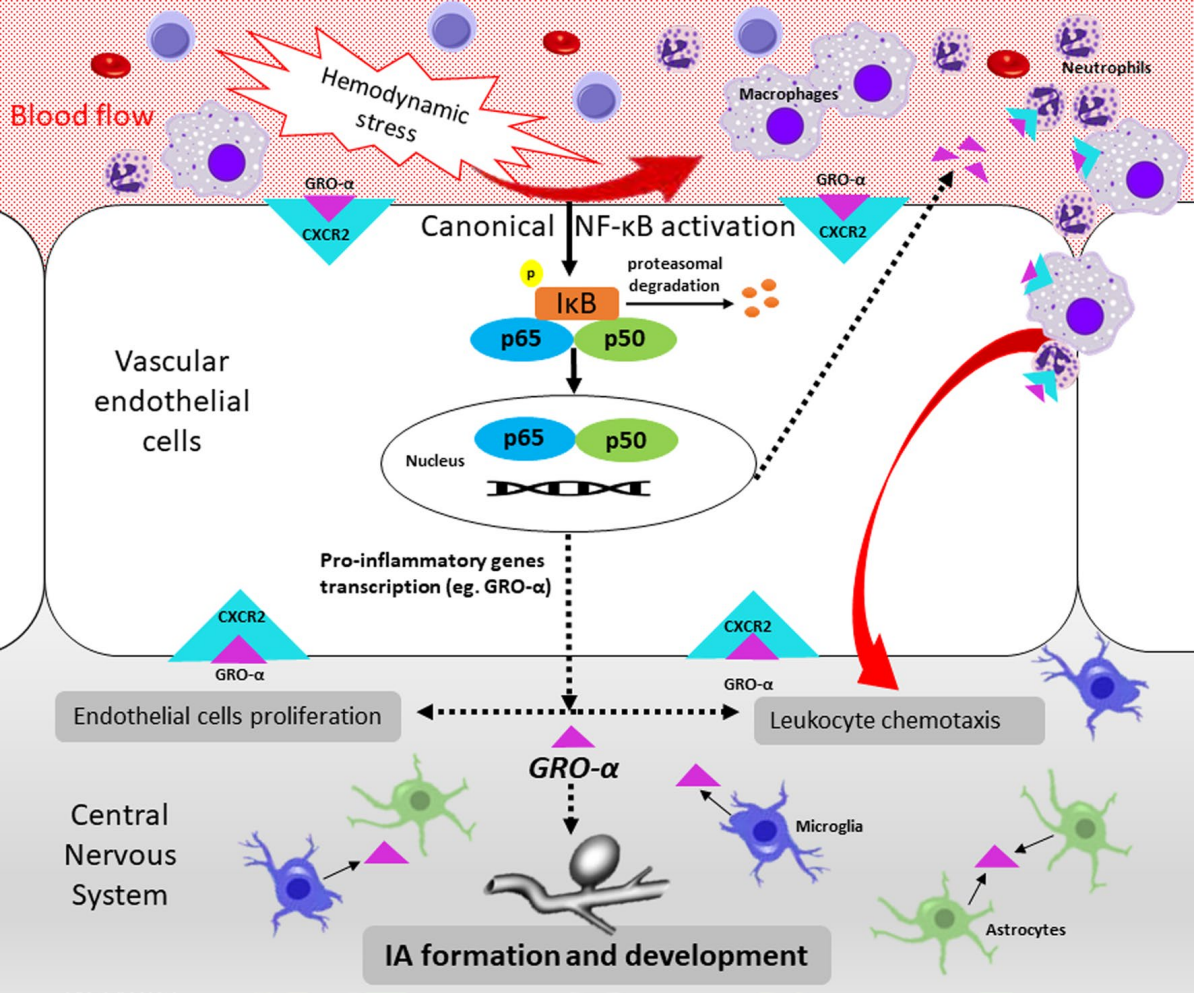
A schematic presentation of the possible role of the NF-κB p65 subunit and the GRO-α/CXCR2 axis in the formation of IA. The first step initiating the formation of IA is the activation of the canonical/classical NF-κB pathway in the arterial bifurcationas as the result of hemodynamic stress. NF-κB activation occurs through the phosphorylation and proteasomal degradation of IκB. An active form of NF-κB p50/p65 heterodimer bind to DNA to function as a transcription factor of pro-inflammatory cytokine genes, including chemokine GRO-α. Secretory chemokine GRO-α, via its CXCR2 receptor, acts as a chemoattractant for leukocytes and increases endothelial cell proliferation, which initiates the formation of IA. As a result of forming IA, also microglia and astrocytes could secrete GRO-α, which in turn enhances leukocyte trafficking and endothelial cell proliferation. CXCR2, C-X-C Motif Chemokine Receptor 2; GRO-α, GRO alpha chemokine/C-X-C motif ligand 1 (CXCL1); IA, intracranial aneurysms; IκB, IkappaB kinase; NF-κB p50, nuclear factor kappa-B p50 subunit; NF-κB p65, nuclear factor kappa-B p65 subunit; NF-κB, nuclear factor kappa-B.

Our study is the first which showed significantly decreased CSF NF-κB p65 and significantly increased CSF GRO-α and its CXCR2 receptor concentrations in UIA patients compared to the control group. These data altogether may suggest that the canonical NF-κB signaling pathway is activated and its target pro-inflammatory genes are highly expressed in UIA patients. The relationship of the proteins tested with the size and number of aneurysms may indicate that the NF-κB p65 pathway and the GRO-α/CXCR2 axis could be potentially involved in the pathomechanism of IA development in humans (Fig. 5). However, to unequivocally assess the involvement of the classical NF-κB pathway with the participation of the NF-κB p65 subunit and the GRO-α/CXCR2 axis in the formation of IA, further in vivo model studies are needed.

## Patients and methods

### Patients

The study was conducted according to the guidelines of the Declaration of Helsinki and the protocol was approved by the Bioethics Human Research Committee of the Medical University of Bialystok (Permission No. R-I-002/383/2015 and APK.002.9.2021). All subjects gave their informed consent for inclusion before they participated in the study.

The study group consisted of 25 patients (5 males/20 females, median age 59 years, range 30–70) with unruptured brain aneurysms (UIA). The patients were operated on at the Department of Neurosurgery at the Clinical Hospital of the Medical University of Bialystok. All patients underwent craniotomy and direct surgical clipping of UIA.

The control group consisted of 10 subjects (3 males/7 females, median age 66 years, range 56–78), suffering from trigeminal neuralgia because of an anatomical conflict between the trigeminal nerve and a cerebellar artery. All patients belonging to this group were qualified for posterior fossa craniotomy and microvascular decompression.

Exclusion criteria for both groups were comprised of: neurodegenerative conditions like multiple sclerosis, neuroinfections and a brain tumor in medical history, surgery or major trauma in the previous months, antibiotics, anti-inflammatory, or corticosteroids administration in the previous months. Detailed information about the study and the control group is described in our previous publication^4^.

### Sample collection and storage

Blood samples were collected in tubes without anticoagulants (S-Monovette, SARSTEDT). Within 30 min after the venipuncture blood was centrifuged for 20 min at 1000 × g to obtain serum, which was further stored at − 75 °C until analysis.

Neurosurgical procedures to obtain cerebrospinal fluid (CSF) were conducted in a standard manner^4^. Particular care was taken to prevent any contamination of the CSF with blood or warm saline solution used as irrigation. All the aforementioned steps were taken at the start of each procedure, before any bleeding may occur. The CSF samples were centrifuged for 20 min at 1000 × g. Obtained CSF supernatant samples were aliquoted and placed in storage at − 75 °C until used.

### Methods

Cerebrospinal fluid (CSF) and serum NF-κB p65, GRO-α, and CXCR2 concentrations were measured using kits based on sandwich enzyme-linked immunosorbent assay technology (ELISA). Samples were not diluted before analysis. Experiments were conducted following the manufacturers’ instructions.

NF-κB p65 concentrations were measured using a human NF-kappaB p65 ELISA kit (Cat#: orb406416) from Biorbyt Ltd., Cambridge, England. The detection range for NF-κB RelA (p65) kit is between 47 and 3000 pg/mL. The assays have high sensitivity and excellent specificity for the detection of human NF-κB p65. No significant cross-reactivity or interference between human NF-κB p65 and analogs was observed. GRO-α concentrations were measured using ELISA Quantikine® Human CXCL1/GROα Immunoassay kit (Cat#: DGR00B) from R&D Systems Europe Ltd., Abingdon, England. The manufacturer of the assay kit referred to the intra-assay coefficient of variation (CV%) as 2.2% at GRO-α mean concentration of 41.8 pg/mL. CSF and serum CXCR2 receptor concentration were measured using a human CXCR2 ELISA Kit (Cat#: orb564834) from Biorbyt Ltd., Cambridge, England. The manufacturer of the assay kit referred to the intra-assay CV% as < 8%, and inter-assay CV% as < 10%.

### NF-κB p65, GRO-α, and CXCR2 Quotients calculation

To exclude the influence of the blood–brain barrier and the blood-CSF barrier on mutual relationships of protein levels in these two compartments^33–36^, we also calculated NF-κB p65, GRO-α, and CXCR2 Quotients by referring to CSF protein value to the serum protein value.

### Statistical analysis

Obtained results were analyzed with the use of the STATISTICA 13.0 PL software (StatSoft Inc., Tulsa, USA) and the GraphPad Prism 8.0 (GraphPad Software, San Diego, USA). Data did not follow the normal distribution (X2-test), thus nonparametric statistical analyses were applied. The Mann–Whitney test was used to compare two independent samples. Correlation coefficients were obtained by applying Spearman’s rank correlation. Receiver operator characteristic (ROC) curves were generated to calculate the areas under the ROC curves (AUCs). To indicate the optimal cut-off points (threshold values) the Youden indexes were estimated. Values for continuous variables are presented as median with 25th and 75th percentiles. Differences were considered significant for two-tailed p < 0.05.

### Ethics approval and consent to participate

The study was conducted according to the guidelines of the Declaration of Helsinki and the protocol was approved by the Bioethics Human Research Committee of the Medical University of Bialystok (Permission No. R-I-002/383/2015 and APK.002.9.2021). All subjects gave their informed consent for inclusion before they participated in the study.

## Supplementary Information

Supplementary Table S1.

## Data Availability

The datasets generated and/or analyzed during the current study are not publicly available, but are available from the corresponding author (JK) on a request.

## Abbreviations

ACC: Diagnostic accuracy
AUC: Area under the ROC curve
CNS: Central nervous system
CSF: Cerebrospinal fluid
CXCR2: C-X-C Motif Chemokine Receptor 2
GRO-α: GRO alpha chemokine/C-X-C motif ligand 1 (CXCL1)
IA: Intracranial aneurysms
NF-κB: Nuclear factor kappa-B
NF-κB p65: Nuclear factor kappa-B p65 subunit
Se: Diagnostic sensitivity
SE: Standard Error
Sp: Diagnostic specificity
UIA: Intracranial aneurysm patients
