# Supplementary material for: Canonical NF-κB signaling pathway and GRO-α/CXCR2 axis are activated in unruptured intracranial aneurysm patients
Source: Sci Rep. 2022 Dec 9;12:21375. doi: 10.1038/s41598-022-25855-2 (PMC9734124; doi:10.1038/s41598-022-25855-2)
Supplement: Supplementary file 1 — Supplementary Table S1. [file 41598_2022_25855_MOESM1_ESM.pdf]

Table S1. Cerebrospinal fluid, serum, and Quotient results for unruptured intracranial aneurysm patients and the control individuals without vascular lesions in the brain.

	Control group N=10	UIA group N=25	2-tailed p-value
	CSF		
NF-κB p65, pg/ml	4.1 (3.7-8.2)	3.6 (3.0-4.2)	0.03
GRO-α, pg/ml	18.5 (14.0-22.9)	26.4 (12.3-38.7)	0.01
CXCR2, ng/ml	0.25 (0.11-0.69)	0.64 (0.47-0.84)	0.03
	Serum		
NF-κB p65, pg/ml	57.2 (19.1-267.3)	8.6 (5.8-58.5)	0.02
GRO-α, pg/ml	14.5 (10.6-28.0)	13.0 (11.7-18.0)	0.69
CXCR2, ng/ml	0.25 (0.11-0.67)	0.17 (0.07-0.23)	0.11
	Quotient		
NF-κB p65	0.07 (0.01-0.22)	0.45 (0.09-0.56)	0.01
GRO-α	1.10 (0.73-1.76)	1.92 (1.13-3.14)	0.04
CXCR2	1.76 (0.62-4.25)	4.17 (2.27-10.25)	0.03

Abbreviations: CSF, Cerebrospinal fluid; CXCR2, C-X-C Motif Chemokine Receptor 2; GRO-α, GRO alpha chemokine; N, number of cases; NF-κB p65, nuclear factor kappa-B p65 subunit; UIA, unruptured intracranial aneurysm. The Quotient was calculated by dividing the CSF protein value by the serum protein value.
